# Computational and Pharmacological Target of Neurovascular Unit for Drug Design and Delivery

**DOI:** 10.1155/2015/731292

**Published:** 2015-10-22

**Authors:** Md. Mirazul Islam, Zahurin Mohamed

**Affiliations:** Pharmacogenomics Lab, Department of Pharmacology, University of Malaya, 50603 Kuala Lumpur, Malaysia

## Abstract

The blood-brain barrier (BBB) is a dynamic and highly selective permeable interface between central nervous system (CNS) and periphery that regulates the brain homeostasis. Increasing evidences of neurological disorders and restricted drug delivery process in brain make BBB as special target for further study. At present, neurovascular unit (NVU) is a great interest and highlighted topic of pharmaceutical companies for CNS drug design and delivery approaches. Some recent advancement of pharmacology and computational biology makes it convenient to develop drugs within limited time and affordable cost. In this review, we briefly introduce current understanding of the NVU, including molecular and cellular composition, physiology, and regulatory function. We also discuss the recent technology and interaction of pharmacogenomics and bioinformatics for drug design and step towards personalized medicine. Additionally, we develop gene network due to understand NVU associated transporter proteins interactions that might be effective for understanding aetiology of neurological disorders and new target base protective therapies development and delivery.

## 1. Introduction

The human brain is one of the most complex organs composed of around 100 billion neurons and glial cells are 10 to 50 times more than the neurons. Major role of neurons cells is to transmit information as electric impulses to other nerve, muscle, or gland cells through special junction called synapse. The brain and central nervous system (CNS) regulate sensory input and motor output as well as coordinating all other body functions. The interface between CNS and the peripheral circulatory system functions as a dynamic regulator of ion balance, a nutrient transport, and molecular trafficking is historically known as blood-brain barrier (BBB) [1]. Although the neurons and the glial cells were the traditional spotlight of neuroscience research, a functional neurovascular unit (NVU) that regulates cerebral blood flow is a comparatively new discipline of study in neurophysiology [2]. The NVU is the integrated system of neuronal and vascular endothelial cells which controls brain homeostasis by restricting the entry of large and harmful molecules [3]. Pharmacological importance of the NVU is gradually increasing given its notion as a successful target for drug designing for prevention or treatment of various diseases [4]. Abnormal function of NVU may cause neurological disorders like Alzheimer's disease [5], Parkinson's disease [6], stroke [7, 8], epilepsy [9], brain tumours [10], trauma [11], and multiple sclerosis [12]. Any proposed novel drugs for the abovementioned diseases would not be effective if they could not penetrate the BBB.

Lok et al. [13] discussed major cell types and cell-cell signalling in the brain related to NVU. McCarty et al. [14] discussed the protein components of NVU (specially integrins) that regulate permeability of BBB. Zlokovic [15] discussed BBB dysfunction and neurodegeneration in Alzheimer's disease. Although Vangilder et al. [16] discussed currently available therapeutics for CNS disease, there are no clear pharmacological or pharmacodynamics approaches of drug targets for pharmaceuticals as well as no upcoming disease prevention strategies. None of the reviews discuss future prospect of target oriented drug design and computational strategy of time saving and cost-effectiveness. In this review we will discuss NVU, regulation of BBB permeability. We also will focus on modern pharmacological approaches for target (NVU) based drug design and efficient delivery as well as recent use of bioinformatics tools for in silico modeling and validation of drugs.

## 2. Blood-Brain Barrier and NVU

**Figure 1:**
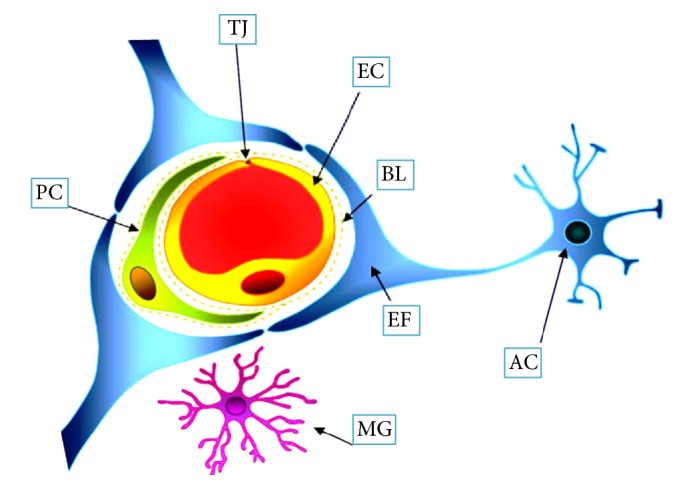
Structure of neurovascular unit. The cerebral capillary endothelial cells (EC) have the tight junction (TJ) that associated with pericytes (PC) and end foot (EF) of astrocytes (ACs). EC and PC are surrounded by basal lamina (BL). ACs are the cellular linker between capillary and neurons. Microglia (MG) are the resident immunocompetent cells of the brain. The movement of solutes either is passive, driven by a concentration gradient from plasma to brain, with more lipid soluble substances entering most easily, or may be facilitated by passive or active transporters in the endothelial cell membranes. This figure is adopted from Abbott et al. [19].

The CNS is the most crucial and sensitive system that needs “gate keepers” to be protected. The BBB is one of the main gate keepers of CNS. It is a dynamic system that plays a vital role for homeostatic aspect of the cerebral microcirculation [1]. The brain capillaries supply blood in proximity to neurons and the brain endothelium forms the largest interface (known as BBB) for molecular and cellular exchange [17]. It is now familiar that the brain endothelium of the BBB acts within a cellular complex recognized as neurovascular unit (NVU) (Figure 1), which is composed of a capillary segment with its associated endothelial cells, pericytes, basement membranes, perivascular astrocytes, neurons, and microglial cells [18].

**Figure 2:**
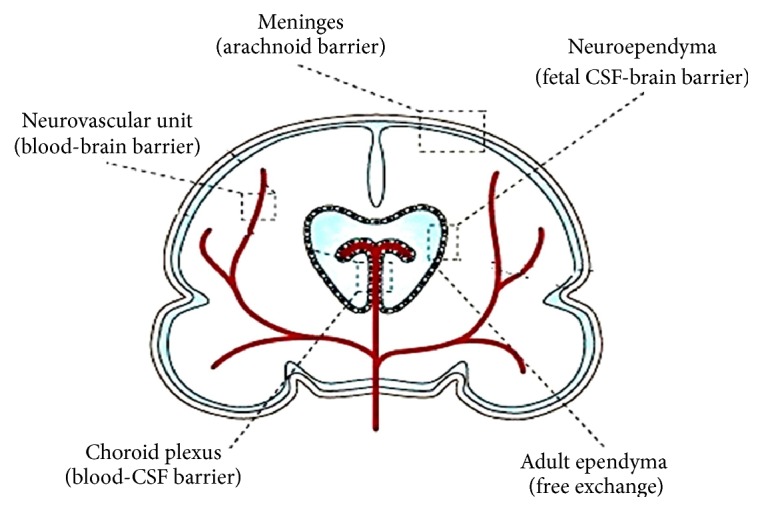
Distributions of barriers in brain. Neurovascular unit, choroid plexus, meninges, neuroependyma, and adult ependyma are the five barriers that regulate dynamic balance between CNS and periphery. This figure is adopted from Neuwelt et al. [31].

The idea of BBB came from variance of dye absorption between periphery and brain [20]. Goldmann injected trypan blue into the cerebrospinal fluid (CSF) of mice and noticed that all brain cells stained but not periphery [21]. So there must be a barrier and the BBB term was first used by Lewandowsky [22]. But the controversy of this invisible barrier was influenced by the observation of basic aniline dyes that crossed the barrier, although acidic aniline dyes did not. Friedemann concluded that electrochemical properties of the molecules determined the permeability of the capillaries of the CNS [23]. Molecular weight, molecular size, binding affinities, dissociation constants, lipid solubility, electric charge, and various combinations of all of these properties determined the rate of entry into the brain [24]. Still some controversy was going on about physical existence of the BBB [25, 26]. This type of controversy is important for scientific progress and new discovery. The issue became clear when electron microscopy (EM) studies could distinguish between capillary lumen and end feet of astrocytes and proposed that interendothelial TJ formed continuous, impermeable membranes that contribute to the formation of BBB [27]. EM suggests the existence of BBB and functional unit of the barrier is considered as NVU. Distribution of the barriers in brain is shown in Figure 2. Later we will discuss how target molecules or drugs cross this barrier and maintain homeostatic balance.

## 3. Cellular Composition of NVU

Highly metabolic and dynamic activities of nervous tissue may have been regulated by blood flow in the brain through BBB, although the cellular mechanisms are not well known [28]. Disruption of BBB integrity is accompanied by neuropathological changes that indicate the selective and compensatory event rather than a simple anatomical disruption [29]. In case of Alzheimer's disease, significant loss of cholinergic innervations of cortical microvessels has been observed that is due to impaired cerebrovascular function [30].

The concept of BBB gradually evolved towards “extended NVU” [31] that formed by astrocytes, pericytes, neurons, microglia, capillary endothelium, immune cells, and the extracellular matrix [3]. Physiological and pathological stimuli of NVU are affected by a complex molecular interaction between cell-cell and cell-extracellular matrix and paracrine cell-cell communication. Regulation of the local cerebral blood flow, BBB permeability and transport mechanisms, neuroimmune responses, and angiogenesis are principal functions that are usually carried out by NVU [3]. Some associated components of the NVU include the circulating blood cells, such as polymorphonuclear cells, lymphocytes, and monocytes that adhere and roll along the vascular lumen and perform surveillance of neural signalling and cellular activity [32]. The CNS of invertebrate and lower vertebrate provides evidence of the evolution of specialized glial cells to pericytes and astrocytes at the vascular-neural interface [33].

Astrocytes are critical in the development and maintenance of BBB characteristics [34] and act as a linker between endothelium and neurons. They also provide tropic influence involved in the moment-to-moment regulation of cerebral microvascular permeability [35] via Ca^2+^ signalling and purinergic transmission [36, 37] as well as functional response of NVU. In vitro culture of brain endothelial cells with astrocytes has been shown to develop BBB characteristics [38]. Pericytes usually located in between end feet of astrocytes and endothelial cells. Presence of some contractile proteins in cerebral pericytes indicates that they may be involved in the regulation of capillary blood flow [39]. Cocultures study indicates the role of pericytes for stabilization of capillary-like structure [40]. In case of hypoxia [41] and traumatic brain injury [42], pericytes have been noticed to migrate away from brain microvessels due to increase BBB permeability. Endothelial expression of occludin (BBB TJ protein) may be induced by pericytes secretion of angiopoietin that indicates the pericytes are involved in induction and maintenance of barrier properties [43].

The extracellular matrix that serves as an anchor for the endothelium also interacts with NVU. Disruption of this matrix is compensated by increased BBB permeability [44]. Matrix proteins can influence the expression of endothelial TJ proteins [45] that indicate the basal lamina proteins are involved in the maintenance of BBB permeability.

In the last decades a significant understanding of NVU associated cells and their molecular and pathophysiological signalling has been developed. This progress is very crucial for understanding neurological disorder and drug development as well as delivery to the brain for the prevention and treatment of the diverse neurological disorder. So, the integrated cellular and molecular concept of the NVU may be implemented in pharmacology and disease understanding that related to cerebral microvascular permeability.

## 4. Molecular Features of NVU

Molecular interactions and signalling usually control the major functions of NVU. Abnormalities of molecular level show physiological change and consequently neurological diseases. Proteins that associated with tight junction of endothelial cells including junctional adhesion molecule- (JAM-) 1, occludin, and claudins have significant role of maintaining permeability. Although JAM-1 is a member of IgG superfamily with large extracellular domain [46] found in epithelial cells, JAM-2 and JAM-3 present in endothelial tissues and lymphatic cells. Occludin has four transmembrane domains that increase electrical resistance of the tissue containing TJ [47]. Paracellular permeability of low molecular proteins increases if C-terminal of the protein is truncated [48]. Claudins have similar membrane topography as occludin [49] and form the primary seal of the TJ; hence occludin acts as additional support structure [1]. Some other signalling molecules like Ca^2+^, cAMP, serotonin, cytokines, chemokines, and steroids may affect BBB permeability. Some endothelial transporters like P-glycoprotein and other proteins of ABC transporter family are potential target for drug design. Ablation of the gene encoding P-glycoprotein leads to defective BBB transport and increased sensitivity to various drugs [50]. As efflux transporter minimizes the drug efficacy by reducing net drug penetration, it could be remodelling target for drug delivery. Hypoxia-inducible factor- (HIF-) 1 is a transcription factor that induced associated gene expression to survive the cell in hypoxic condition [51]. Vascular endothelial growth factor (VEGF) secreted by astrocytes during inflammatory response may induce angiogenesis with the help of other adhesion molecules [52]. Proangiogenic NVU remodelling factors could be potential and considerable target for future drug design and delivery. So the remodelling of NVU during hypoxia may cause profound change of BBB function and this strategy could be useful for drug delivery by creating artificial short-term adjustment.

**Figure 3:**
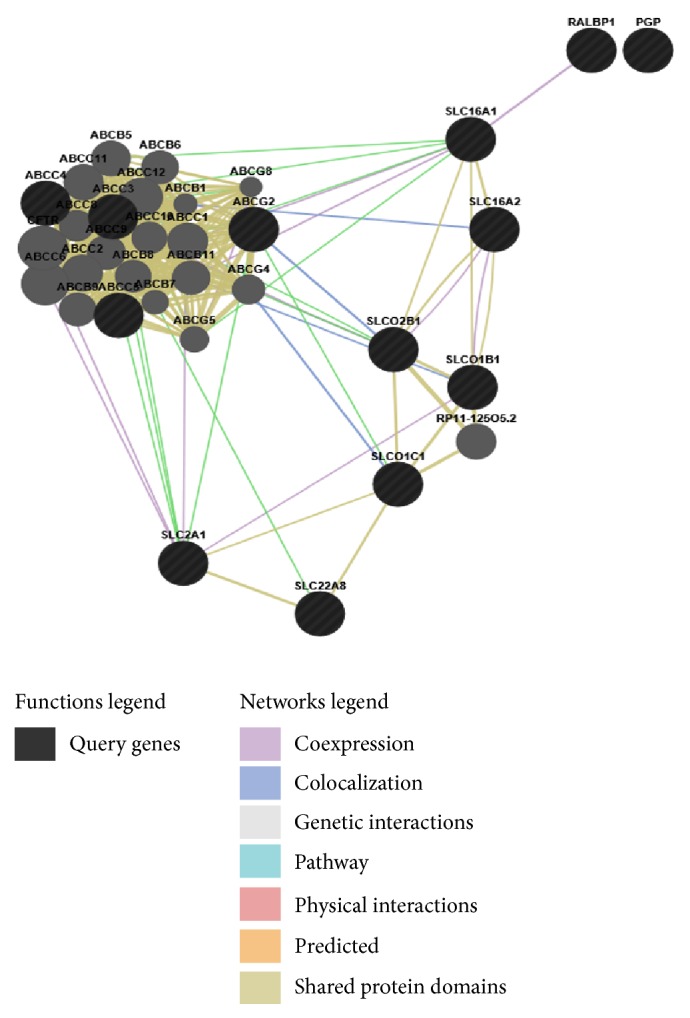
BBB transporter gene network. Gene network constructed with the help of GeneMANIA [54] tools. Black circles represent the transporter genes and gray circles represent the genes that related to the transporter genes. SLC gene family is actively linked to BBB transportation.

We have created a network (Figure 3) of 13 different genes using GeneMANIA [53] which encode transporter protein related to NVU.

## 5. Functional Characteristic of NVU

**Figure 4:**
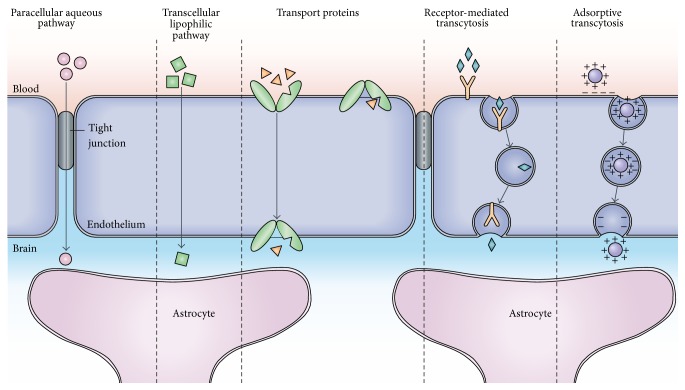
Pathways across the blood-brain barrier. Endothelial cells and end feet of astrocytes that form NVU contain five trafficking routes. Only a handful of drugs can cross BBB. Although most CNS drugs enter via lipophilic rout (size no longer than 600 Da), pharmacological targets are another three pathways to efficient drug delivery. The figure is adopted from Abbott et al. [59].

NVU has dynamic function to regulate BBB permeability in the brain. CNS homeostasis is maintained by complex transport mechanisms that adjust the balance between influx of nutrients and efflux of wastes, toxins, and drugs [55]. Various factors regulate the barrier permeability of the NVU, including membrane transporters and transcytotic vesicles (Figure 4) [56]. Recent understanding of ion transporter proteins and their role of fluid balance and water and electrolytes movement by the cells of NVU has been expanded [31]. Presence of aquaporins in astrocytes end feet [57] indicates their role in fluid dynamics and pathological consequences may occur due to malfunction [58].

Xenobiotics resistance to brain is another important function of NVU maintained by two components of endothelial cells: efflux transporters and tight junctions [31]. P-glycoprotein (Pgp) transportation is regulated by efflux transporters. Paracellular diffusion of water-soluble solutes and drugs, from blood to brain, is restricted by tight junctions [40].

Signalling between neurons and astrocytes may influence cerebral blood flow [60]. Astrocytic calcium signalling triggered the vasoactive messengers [37] that alter local cerebral blood flow. Pericyte-endothelial cell interactions regulate some properties of the BBB during development, and disruption of these interactions may lead to BBB dysfunction and, thus, to neuroinflammation as part of the response to CNS injury as well as consecutive diseases [61].

## 6. Disease Aetiology of NVU Abnormalities

Abnormal NVU selective permeability function may cause various CNS diseases (Table 1). The capillaries of brain tumours are more leaky than normal brain tissue [62]. During the process of aging, several vascular risk factors including hypercholesterolemia, hypoglycemia, and hypertension damage the NVU leading to chronic hypoperfusion, BBB dysfunction, and common NVU pathophysiological responses [63]. Alzheimer's disease (AD) patients have more strokes than age-matched controls [64] that indicate a correlation between NVU disorder and AD. VEGF disrupt TJ and increase BBB permeability. Migrating endothelial cells and pericytes release metalloproteases that may disrupt basement membrane [3]. Influx of serum proteins (albumin, thrombin) and water (through the disrupted membrane) is responsible for liquid accumulation that causes oedema and triggers astrocytic response. Some recent data suggest that acute increase of BBB permeability changes the extracellular ionic environment that promotes high synchronicity and excitability of neuronal network [65, 66] and contributes to glial immune response that might be a causative agent of epilepsy.

## 7. Diagnostic and Therapeutic Target of NVU

There are sufficient evidences that BBB disruption is the early event in many neurological disorders; growing interest of therapeutic target on that region is not unusual [75, 76]. Acute neuroinflammatory impact of BBB due to traumatic brain injury has substantial role in drug targeting, as some animal studies [77, 78] already found the initiation of transcriptional changes in neurovascular network for BBB breakdown that leads to neurodegeneration [79].

Whether we could stop neurodegeneration by inhabiting inflammatory cytokines is the next focus of study. Anakinra is the inhibitor of interleukin 1 (IL-1), successfully used for rheumatoid arthritis treatment [80]. Nonsteroidal anti-inflammatory drugs (NSAIDs) are another successful example based on inhibitory mechanism that is being used for Alzheimer's disease [81]. So, we can say that BBB disruption based neuroinflammation could be a potent therapeutic target.

As varieties of neurological diseases are linked to NVU, continuous research efforts might lead to identifying specific biomarkers and to developing therapeutic strategies to control the abnormalities. Due to accessibility and expression in early stage, vascular compartment specific molecular biomarkers are attractive and can be detected in situ using molecular imaging or in the circulating compartment using “omics” approaches [3]. Angiogenic brain tumour can be identified by microarray analysis of specific markers [82]. Monoclonal antibody based treatment of multiple scleroses which binds the *α*4 integrin receptor found on leukocytes that prevents adhesion of the leukocytes to brain endothelial cells [83]. Protection of BBB integrity by inhibiting metalloprotease could be therapeutic treatment of stroke [84].

Combination of L-DOPA and carbidopa is the potential drug used for Parkinson's disease but bioavailability of oral dose is less than 1% due to efflux pump [1]. Donepezil is acetylcholinesterase inhibitor that can treat Alzheimer's disease, but P-gp mediated efflux limits therapeutic concentration [85]. Refractory epilepsy does not respond to antiepileptic drugs because of increased expression of P-gp and multidrug resistance efflux pumps [86]. Brain cancer treatment is also difficult for limited accessibility of chemotherapeutic agents to BBB.

Dexamethasone is being used for brain oedema that regulates P-gp expression and constrains TJ in brain endothelial cell [87]. Differential display technique gives us the idea of disease causing gene expression that could be target for therapy [88]. A gene network of transporter protein is already shown in Figure 3. Intraventricular administration of VEGF increases endothelial permeability (possibly involving activation of the PI-3-Akt pathway) that might act through BBB associated cells [89]. So, we can easily say that NVU is not the only emerging area; associated capillary endothelial cells of BBB also could be future diagnostic and therapeutic target of neurological disorders [90].

## 8. Modern Pharmacological Approaches of NVU Based Drug Delivery

A majority of well-known drugs give only symptomatic relief for a limited period with adverse side effect and toxicity. Around 98% of small drugs and nearly 100% of large drugs molecules cannot penetrate the brain in sufficient therapeutic amount [91]. There is no meaning of a drug if it cannot be transported across the BBB.

Prodrugs like L-DOPA could penetrate through carrier mediated transport when targeting endogenous transport protein. Molecular Trojan horses use receptor-mediated transcytosis (RMT) that could permit large molecule to the brain [92]. Insulin receptor could be a better candidate for drug delivery with human insulin monoclonal antibodies which works 900% more actively and 10 times more effectively in comparison to transferring receptor [93]. But efflux pumps of astrocyte have limited squeezing efficacy in case of larger molecules [94]. Liposomes are good drug transporter and enhance bioavailability of the drug. DepoFoam is an advance liposome that performs continuous release of drugs with noninvasive strategy. This technique poses a great potential for CNS drug delivery with lower dosing and better efficacy. Although fewer side effects have been reported, none of those were severe [95]. Insulin, lipoprotein, and diphtheria toxin receptors are potential for molecular delivery in brain. Another relatively new approach is cell mediated transcytosis that is considered the outcome of rapid progress of molecular biology. Gene therapy is another molecular technique that could replace disease causing gene in the cell. Transport vectors could activate natural transport rout and enhance entry to the brain. Clinical use of gene therapy is still limited due to immunogenic safety issue [96]. So, ion channels, neurotransmitters, growth factors, and transcription factors are prospective therapeutic targets for new drugs delivery.

Our recent understanding of cellular receptor and polymer chemistry brings a new field of drug delivery called nanotechnology. This nanoparticle could be transported in various parts of body and deliver drugs including brain [97]. Modified surface properties of the particle can carry different types of drugs to different part of our body. One of the important components of nanoparticles is Human Serum Albumin (HSA) that can be modified and works without serious side effect [98]. Some nanoparticle based cancer drug delivery is still in clinical trial. In spite of being a potential field of drug delivery, a handful of nanoparticles are preclinical evaluation due to small size, aggregation, and physical handling difficulties [99]. However, there might be many threatening possibilities for especially long-term impact of such drugs and metabolism of the particle should be considered carefully.

## 9. Computational Approach of Drug Design and Delivery

New drugs development and increase of the existing drugs efficacy are the major challenges of pharmacology. Traditional drug designing protocol is time-consuming, risky, and indeed costly. Katara [100] described the role of bioinformatics and pharmacogenomics for drug design but not BBB oriented drug delivery. We integrate the recent approaches of target based drug. Computational biology is a new field that emerged in the last decades and integrated to pharmacology due to assist drug design and delivery. We cannot think about personalized medicine without another increasing branch called pharmacogenomics [101].

Recently P-gp is being used as therapeutic target for optimizing CNS drug delivery that is based on pharmacogenomics data analysis [102]. Pharmacogenomics refers to the effects of single nucleotide polymorphism (SNP) and copy number variation (CNV) on drug response; its knowledge can help in selection of the optimal drug, dose, and treatment process and avoid adverse drug reactions [103]. The PharmGKB is a pharmacogenomics knowledge resource that comprehends clinical information, clinically actionable gene-drug associations, and genotype-phenotype relationships [104]. Integration of this entire branch can accelerate various steps of drug designing and reduce the time as well as overall cost.

Constant pressure of generating various drugs within limited time period with low risk has resulted in remarkable interest in bioinformatics [105]. The major benefits of bioinformatics are to sort out the biologically active and potential candidates and predict and identify their biological phenomena using data mining [106]. Human genome project gives us the available public data [107] for mining and generating valuable data for computer-aided drug design and delivery approaches that greatly increase the potential drugs candidates in the pipelines of pharmaceutical companies [105, 106]. Store and analysis of those huge data are not easy and computer scientists bring out a solution called cloud computing. Several drug target databases are available online. DrugBank is a comprehensive drug information database that works based on bioinformatics and chemoinformatics data [108]. SuperTarget is a 2D drug screening and sequence comparison database for the extensive drug target [109]. Search Tool for Interactions of Chemicals (STITCH) and Ingenuity Pathway Analysis (IPA) are the searchable database that summarizes information from text mining, metabolic pathway, drug target relation, and structural similarity [110]. Therapeutic Target Database (TTD) is a therapeutic target database that provides information of known therapeutic protein and nucleic acid [111].

In spite of drug target validation, bioinformatics provides different algorithms that reduce the failure of clinical trial approaches [112]. Physiology of experimental lab animals and human being is not the same which could be a notable burden for the final stage of clinical trial. Computational biology gives us the in silico validation and docking analysis opportunity before further step.

We have the genomic data and we know the cellular composition of NVU as well as transporter proteins. So we could in silico validate the drug candidates regarding efficacy to brain and accessibility to BBB. TJ is the main regulator of NVU permeability and we know the structural composition of TJ associated proteins. How we could regulate and adjust the short-term opening of TJ is a burning question of drug delivery to brain. Also there is extensive study area of surrounding cell physiology and molecular function that might give us details and integrated view.

Pharmaceutical companies are focused on blockbuster drugs prescribed to more than 20 million people. Recent Ebola outbreak in Africa shows us the necessary of orphan drug development. This raises an issue for developing countries and bioinformatics could be the possible hope for the orphan drug development as per need [113].

Nowadays it is quite easy to identify drug candidate and target using online database but experimental validation is not easy. Although bioinformatics is a potential field, it did not bring any considerable change yet for drug design and delivery process. This is due to less acceptance of new technology and lack of technical expertise. Also changing the traditional drug market is a big issue for the pharmaceutical companies and investors. We can expect that in the near future the situation will change and bioinformatics based drug design and delivery through NVU will be popular and time- and cost-effective with high level of drug efficacy.

## 10. Conclusions

We still have limited knowledge of the human brain and most of its functions that remain unknown or mysterious. This paper has described the role of NVU in CNS homeostasis and potential target for therapies. Bioinformatics and pharmacogenomics can provide huge support for pharmaceutical companies in order to design drug for neurological disorder with reasonable time and affordable cost. Drug trials might have some limitation in order to measure the functionality of therapeutics. We need to minimize the gap between cell culture and animal model study to get appropriate understanding of drug delivery. Application of molecular biology into neuroscience could help to understand genetic make-up, epigenetic variation, and near prospect of personalized medicine. Integrated system biology approaches could help to know insights into disease aetiology, progression, and target oriented cocktails drugs design. Although bioinformatics and pharmacogenomics are passing initial phase of development, they already pose enough potential for future drug industry. Brain targeted drug delivery must be safe and beneficial for patients and have to ensure minimum short- or long-term impact. Finally, it can be said that NVU would be the main target of pharmacokinetics to reduce drug abuse and to answer some unsolved questions of neuroscience.

## Figures and Tables

**Table 1:** Pathological consequences of NVU disorder.

Diseases	BBB proteins and affected mechanisms
Alzheimer's disease	BBB disruption and permit peripheral IgG to brain. Decrease P-gp and accumulate amyloid-*β* in brain [67].

Parkinson's disease	BBB disruption increases therapeutic agent concentration and reduces efficacy of Pgp [6].

Stroke	Astrocytes secrete TGF*β* that downregulates tissue plasminogen activator (tPA) and anticoagulant thrombomodulin (TM) [68].

Epilepsy	Transient BBB opening and upregulation of multiple drug resistance (MRD1) Pgp [69].

Trauma	Opening of BBB, release of IL-6 from astrocytes, and neuroinflammation [70].

HIV	BBB TJ disruption. Loss of glycoproteins and apoptosis of endothelial cell lead to increase diameter of cortical vessels [71].

Infectious processes	Increase CSF/serum albumin ratio. Bacterial lipopolysaccharides affect BBB TJ [72].

Brain tumours	Breakdown of BBB TJ, overexpress folate, insulin, and transferrin receptor, and downregulation of claudin 1/3 [73].

Ischaemic brain oedema	BBB breakdown due to MMP9 release by neutrophils and degradation of occludin, claudins, and JAM [74].
